# An international cross-sectional survey on the Quality and Costs of Primary Care (QUALICO-PC): recruitment and data collection of places delivering primary care across Canada

**DOI:** 10.1186/s12875-015-0236-7

**Published:** 2015-02-18

**Authors:** Sabrina T Wong, Leena W Chau, William Hogg, Gary F Teare, Baukje Miedema, Mylaine Breton, Kris Aubrey-Bassler, Alan Katz, Fred Burge, Antoine Boivin, Tim Cooke, Danièle Francoeur, Walter P Wodchis

**Affiliations:** School of Nursing, University of British Columbia (UBC), Vancouver, Canada; Centre for Health Services and Policy Research, University of British Columbia (UBC), Vancouver, Canada; Department of Family Medicine, University of British Columbia (UBC), Vancouver, Canada; The Canadian Primary Health Care Research and Innovation Network (CPHCRIN), Ottawa, Canada; Department of Family Medicine, University of Ottawa, Ottawa, Canada; C.T. Lamont Primary Health Care Research Centre, Élisabeth Bruyère Research Institute, Ottawa, Canada; Institute of Health Policy Management and Evaluation, University of Toronto, Toronto, Canada; Toronto Rehabilitation Institute, Toronto, Canada; Institute for Clinical Evaluative Sciences, Toronto, Canada; Saskatchewan Health Quality Council, Saskatoon, Canada; Faculty of Medicine, Dalhousie University, Halifax, Canada; Family Medicine Teaching Unit, University of New Brunswick, Fredericton, Canada; Université de Sherbrooke, Charles LeMoyne Research Center Hospital, Sherbrooke, Canada; Faculty of Medicine, Memorial University of Newfoundland, St. John’s, Canada; Health Sciences Centre, St. John’s, Canada; Departments of Community Health Sciences and Family Medicine, University of Manitoba, Winnipeg, Canada; Manitoba Centre for Health Policy, Winnipeg, Canada; Department of Family Medicine, University of Montreal, Montreal, Canada; Health Quality Council of Alberta, Calgary, Canada; Institut national de santé publique du Québec (INSPQ), Québec, Canada

**Keywords:** Primary health care, Physician, Response rate, French, English, Survey, Practice

## Abstract

**Background:**

Performance reporting in primary health care in Canada is challenging because of the dearth of concise and synthesized information. The paucity of information occurs, in part, because the majority of primary health care in Canada is delivered through a multitude of privately owned small businesses with no mechanism or incentives to provide information about their performance. The purpose of this paper is to report the methods used to recruit family physicians and their patients across 10 provinces to provide self-reported information about primary care and how this information could be used in recruitment and data collection for future large scale pan-Canadian and other cross-country studies.

**Methods:**

Canada participated in an international large scale study-the QUALICO-PC (Quality and Costs of Primary Care) study. A set of four surveys, designed to collect in-depth information regarding primary care activities was collected from: practices, providers, and patients (experiences and values). Invitations (telephone, electronic or mailed) were sent to family physicians. Eligible participants were sent a package of surveys. Provincial teams kept records on the number of: invitation emails/letters sent, physicians who registered, practices that were sent surveys, and practices returning completed surveys. Response and cooperation rates were calculated.

**Results:**

Invitations to participate were sent to approximately 23,000 family physicians across Canada. A total of 792 physicians and 8,332 patients from 772 primary care practices completed the surveys, including 1,160 participants completing a Patient Values survey and 7,172 participants completing a Patient Experience survey. Overall, the response rate was very low ranging from 2% (British Columbia) to 21% (Nova Scotia). However, the participation rate was high, ranging from 72% (Ontario) to 100% (New Brunswick/Prince Edward Island and Newfoundland & Labrador).

**Conclusions:**

The difficulties obtaining acceptable response rates by family physicians for survey participation is a universal challenge. This response rate for the QUALICO-PC arm in Canada was similar to rates found in other countries such as Australia and New Zealand. Even though most family physicians operate as self-employed small businesses, they could be supported to routinely submit data through a collective effort and provincial mandate. The groundwork in setting up pan-Canadian collaboration in primary care has been established through this study.

## Background

In Canada, performance reporting in primary health care (PHC) is challenging because of the dearth of concise and synthesized information [1]. The paucity of information occurs, in part, because the majority of PHC is publicly funded through a single payer (i.e. provincial and federal governments) but delivered through a multitude of privately owned small businesses [2], also known as PHC practices. The majority of PHC practices are family physician owned and operated; they employ staff, pay overhead, and provide health care services to patients. The majority of family physicians’ income is derived from billing the government fees for services, which are negotiated between each provincial government and organized medicine in their respective jurisdictions, with some being paid through a blend of payments [3]. Many family physicians are part of a group or interprofessional practice, such as group practices or community health centres, while others provide care in a solo practice [3].

Outside Canada, reporting on PHC performance also remains challenging [4-7]. Indeed, the activity of reporting in PHC is not a traditional role providers are accustomed to. Despite PHC being publicly funded in developed countries, providers have not considered it their role to report on their performance or the performance of their practice with the goal of improving the PHC sector or larger health care system.

Yet, there are growing demands for performance reporting from many stakeholders including patients [8,9]. Regional case studies of performance reporting [10,11] and evidence from the hospital sector [12] indicate it can influence quality improvement agendas and improve performance. Past work shows that public reporting may improve performance [6,10,13-15], as it has the potential to “improve the quality of care, increase accountability, facilitate public participation in health care,”([14], p.62, 15) impact societal and professional values and direct attention to issues not currently on the policy agenda [16-18]. It may also facilitate collaboration among stakeholders as they set a common agenda [19]. While performance reporting in the hospital sector grows, performance reporting in PHC lags behind.

The most commonly referenced performance information about PHC internationally is from the Commonwealth Fund patient and clinician surveys in industrialized nations [20-26]. The surveys are based on samples of 1000 patients or clinicians per country and show (for the dimensions addressed by the surveys) that PHC performance in Canada is poor compared to other Organization for Economic Co-operation and Development (OECD) countries. In an attempt to improve the quality of information used to report on PHC performance, the international QUALICO-PC (Quality and costs of primary care) study was developed with the overarching goals of: 1) examining the relationship between the strength of the primary care system and the performance of the overall healthcare system ([27] and 2), satisfy the demand for benchmarking performance information and to inform primary care reform through cross national comparisons [27,28]. The purpose of this paper is twofold: 1) to report the methods used to recruit family physicians and their patients across the 10 provinces of Canada to participate in the QUALICO-PC study; and 2) to interpret patterns of recruitment to participate in this study. This work is important in reflecting on what could be done in recruitment and data collection for future large scale pan-Canadian and other cross-country studies.

## Methods

### Design

The QUALICO-PC study started as a research program funded by the European Union (EU) including 26 member states and five non-EU European countries, Iceland, Macedonia, Norway, Switzerland, and Turkey [27,28]. Outside Europe, Canada, Israel, Australia and New Zealand also participated, funding their own participation. A total of 34 countries participated. QUALICO-PC used a cross-sectional study design to collect self-reported data from family physicians, their practices and 10 patients who were seen by them.

### Survey content

A set of four surveys, designed to collect in-depth information regarding primary care activities was collected [27]. The surveys, which are described in detail elsewhere [27]. included concepts important to the delivery and organization of primary care through individual patient, physician, and practice surveys. The practice survey (PRA) collected information on organizational features such as design and delivery of primary care (e.g., financing, regulation, resources), whereas the family physician survey (FPS) collected information on the type of tasks and services (e.g., first contact care, prevention, continuity of care, and integrated service provision) delivered. The patient experiences survey (PES) contained questions aimed at collecting information on their experiences including: coordination, continuity, quality of care, and equity in treatment of primary care. The patient values survey (PVS) asked questions on the importance of access, quality of care (e.g. interpersonal communication) and services delivered in primary care. The surveys and procedures for collecting the data were originally developed and validated by the European team [29]. Minor adjustments were made by the Canadian research team to align with the different health care systems of the provinces, yet remained as close as possible to the validated European surveys.

### Eligibility of participants

All 10 provinces in Canada participated in QUALICO-PC; Canada’s provinces vary in geographic size and population, with Québec, Ontario, and British Columbia being the largest provinces in terms of land mass and population. Prince Edward Island is Canada’s smallest province in terms of land mass and population. Two of Canada’s smaller provinces, New Brunswick and Prince Edward Island, combined their recruitment and data collection efforts. Physicians who were working with a family/general practice (e.g., not specializing in a narrow set of conditions or treatments) were eligible to participate. We maximized recruitment of the variety of practices where family physicians work by having only one physician per practice eligible to participate. A "practice" was defined as one or more physicians that share one of revenue, staff or patients. Only patients of participating family physicians were eligible to take part in the study. Patients had to be 18 years of age or older, speak and read English or French, and not have cognitive impairment to participate.

### Sources and methods of participant selection

Each provincial team followed the same data collection method but recruitment methods varied slightly from province to province. For example, in Alberta, a notice of the study was posted on both the Alberta Medical Association and Alberta College of Family Physicians websites, and all related survey material was posted on the Health Quality Council of Alberta site for physician reference.

All family physicians were recruited in collaboration with organizations that had lists of practicing physicians such as the provincial chapters of the Canadian College of Family Physicians. All provinces, except Québec and Manitoba, used a census approach where all family physicians on these organizations’ lists were recruited to be study participants. Some provinces (Manitoba, New Brunswick and Prince Edward Island) worked with organizations who could recruit from their membership, while other provinces (Québec) received permission to use a list of primary care physicians registered to the Department of Health or the Fédération des médecins omnipraticiens du Québec. The research lead and provincial chapters of the Canadian College of Family Physicians or other authorities (e.g., Departments of Family Medicine) gave their support in jointly inviting family physicians to participate in the Canadian QUALICO-PC study through mailed or emailed letters. Interested physicians registered either online or by fax. The Québec recruitment differed in that physicians were called and could register over the telephone if they did not respond by mail or email. Additionally, Québec specifically tried to recruit a stratified random sample of physicians by geographic area and, in some cases, allowed participation by more than one family physician from each practice to accommodate geographic areas with a lower number of practices. In Manitoba recruitment was weighted so that 67% of the family physicians were in Winnipeg; the rest were randomized from non-Winnipeg regional health authorities to try and ensure equitable representation.

### Procedures

The Canadian Primary Health Care Research and Innovation Network http://www.cphcrin-rcrissp.ca/ coordinated efforts across the 10 provinces [30]. They facilitated the provincial research teams in meeting regularly throughout the recruitment phase. This pan-Canadian working group was responsible for planning and coordinating data collection, data sharing, and analyses of the Canadian data [31]. An underlying principle during the recruitment phase of this working group was to minimize potential bias in recruitment by creating a supportive learning environment through sharing ethics applications and recruitment materials and mutual problem solving. As much as possible data collection followed a standard protocol and was led by one of the research team members from that province.

Family physicians and patients were required to provide informed consent. In some provinces, personal health numbers (PHNs) and full date of birth were collected from consenting patients for linkage to administrative databases in order to determine relationships between physician, practice, patient experiences/values, utilization of medical services and health outcomes. Family physicians were compensated $200 CDN as a token of recognition for the disruption to their work.

Data were collected in 2013 and early 2014; Once data collection started in each province, it lasted up to 4 months except in Québec where data collection lasted for 9 months. Physicians who registered were contacted by a research assistant by telephone or email to confirm eligibility and interest in participation. Each participating physician was couriered a package containing Scantron [32] format surveys, instructions, pens, and return courier packages. In addition to filling out the provider (n = 1) and practice (n = 1) surveys, physicians were asked to choose a day during which the patients to be seen represented their regular patient panel. Patient surveys (n = 9 patient experience; n = 1 patient values) were administered to consecutive consenting patients on this day. If the surveys were not completed in a single day, recruitment continued for up to 3 days. Practices couriered back their completed surveys. Practices that registered (online, telephone, fax, or email) were contacted up to a maximum of 21 times to encourage completion and return of their surveys. It is important to note in cases where there were a higher number of contacts, this was sometimes due to the research team and family physician office leaving messages for each other, the office asking the research team to call back, or that the family physician was on holidays.

De-identified survey data were scanned into a data file by the provincial research team or couriered to Canmark Technologies Ltd. [33], a third party purveyor located in Toronto, Ontario. The provincial data files were subsequently merged into a national dataset for cross-jurisdictional comparisons. Any hard copies of the surveys originally couriered to Canmark were couriered back to the provincial sites to be stored in a secure location. Separately, and only within each provincial research team, the personal health numbers were entered along with the study ID number into a secure encrypted file. All procedures were approved by behavioural research ethics boards (BREBs) located at the institution where each provincial lead investigator was affiliated (BREB number for lead author’s institution: H12-03300). See Table 1 for the names of the research ethics boards.

**Table 1:** **Research Ethics Boards**

**Province**	**Institution**	**Ethics board**
British Columbia	University of British Columbia	Behavioural Research Ethics Board
Alberta	Health Quality Council of Alberta	Community Research Ethics Board of Alberta
Saskatchewan	Health Quality Council of Saskatchewan	University of Sakatchewan Behavioural Research Ethics Board
Manitoba	University of Manitoba	Health Research Ethics Board
Ontario	University of Toronto	Health Sciences Research Ethics Board
	University of Ottawa	Health Sciences and Science Research Ethics Board
Québec	University of Sherbrooke	Comité institutionnel d'éthique de la recherche avec les êtres humains
New Brunswick/PEI	Horizon Health Network	Research Ethics Board
Nova Scotia	Dalhousie University	Health Sciences Research Ethics Board
New Foundland & Labrador	Memorial University	Interdisciplinary Committee on Ethics in Human Research

In Canada, we followed the protocol devised by our European counterparts. The target sample for European countries was generally 220 family physicians from 220 different practices per country with a few smaller countries aiming for samples of 75 family physicians from 75 different practices. In order to compare across provinces, our target sample was 220 family physicians from different practices in three of the most populous provinces (Alberta, Ontario and Québec) and 75 family physicians from different practices in all other provinces (with New Brunswick and Prince Edward Island combined as a single sampling unit).

### Data management and analysis

Using an excel spreadsheet, each provincial team was asked to keep records on the number of: physician recruitment emails/letters sent, physicians who registered, practices who were sent surveys, and practices who returned the completed set of surveys. Data were also recorded on the number of contact attempts made to practices to encourage their participation. Finally, field notes were recorded by each provincial team to gain an understanding of recruitment challenges and reasons why practices were unable to participate. All recruitment data were managed by one research assistant in British Columbia.

*Response* rates were calculated as the number of physicians who signed up to participate in the study divided by the total number of invitations sent out. *Participation* rates were calculated as the number of physicians who were sent a package of surveys divided by the number of physicians who signed up to participate in the study. *Cooperation* rates were calculated as the number of physicians who returned completed surveys divided by the total number of physicians who received surveys. Field note data, including reasons why practices were unable to participate were aggregated into themes. Credibility and trustworthiness [34] of the data were undertaken by having the provincial teams discuss the resulting themes and also through international [35] and national [36] dialogue.

## Results

Invitations to participate were sent to approximately 23,000 family physicians across Canada. The majority of invitations were sent to family physicians in Ontario, followed by British Columbia, and Alberta. The frequency of invitations (including initial) sent ranged from 2–4 across the provinces. The frequency of follow-up phone calls to encourage registered physicians and their practices to complete data collection and return their packages ranged from 1–21. The mean number ranged from 3.3 in Newfoundland and Labrador to 6.53 in Québec.

Across Canada a total of 792 physicians and 8,332 patients from 772 primary care practices in Canada completed the surveys, including 1,160 participants who completed a Patient Values survey and 7,172 participants who completed a Patient Experience survey (Table 2). Patient participants who also consented to link their survey data to administrative data ranged from 57% in New Brunswick/Prince Edward Island to 86% in British Columbia.

**Table 2:** **QUALICO-PC completed surveys by province**

**Completed surveys**	**British Columbia**	**Alberta**	**Saskat chewan**	**Manitoba**	**Ontario**	**Québec**	**New Brunswick/PEI**	**Nova Scotia**	**New Foundland & Labrador**	**Total completed surveys**
Physician surveys, n	59	116	20	41	184	218	54	59	41	792
Practice surveys, n	58	117	20	24	183	218	53	58	41	772
# Patient experience surveys, n	537	1240	185	353	1698	1798	497	544	320	7,172
# Patient values Surveys, n	90	207	33	48	282	289	69	92	50	1,160
**Total surveys by province**	744	1680	258	466	2347	2523	673	753	452	

**Figure 1:**
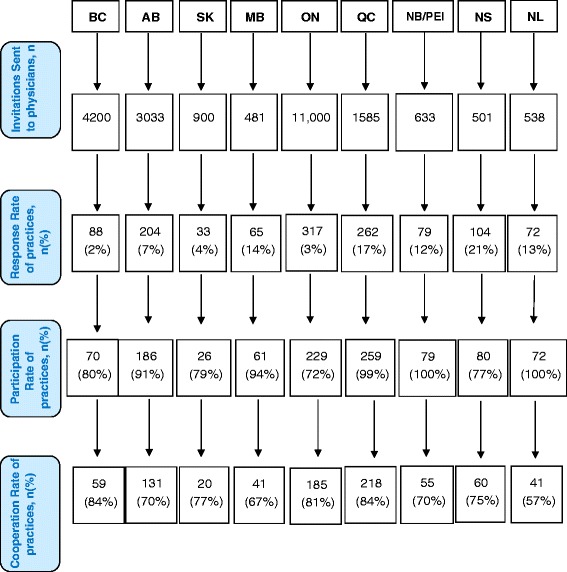
**Flow diagram: Pan-Canadian QUALICO-PC Recruitment.** Response rates were calculated as the number of physicians who signed up to be part of this study divided by the total number of invitations sent out. Participation rates were calculated as the final number of eligible physicians who were sent a package of surveys divided by the number of physicians who signed up to participate in the study. Cooperation rates were calculated as the number of physicians who returned completed surveys divided by the total number of physicians who received surveys. Abbreviations: British Columbia (BC), Alberta (AB), Saskatchewan (SK), Manitoba (MB), Ontario (ON), Québec (QC), New Brunswick/Prince Edward Island (NB/PEI), Nova Scotia (NS), Newfoundland and Labrador (NL).

Four sites across five provinces, Ontario, Québec, New Brunswick/Prince Edward Island, and Nova Scotia (see Figure 1, response rate) met their targeted sample size of practices through the recruitment procedures. Overall, the response rate was very low ranging from 2% (British Columbia) to 21% (Nova Scotia). Even with a longer data collection period, Québec achieved only an 18% response rate. However, the participation rate was high, ranging from 72% (Ontario) to 100% (New Brunswick/Prince Edward Island and Newfoundland & Labrador). The cooperation rate (see Figure 1) shows that although no province successfully collected data from the targeted number of practices, once practices agreed to participate and received the data collection package, the majority of them were able to return completed surveys. The cooperation rate ranged from 57% (Newfoundland and Labrador) to 84% (British Columbia and Québec).

Across all provinces except New Brunswick/Prince Edward Island and Newfoundland, there was some attrition that affected the participation rate (n = 162). In confirming eligibility, 65 (40%) of the family physicians were found to be located in the same practice as one of their colleagues who had already been admitted to the study. In some cases, one of the team members was unable to reach the physician who had signed up online (n = 32). In other cases, once data collection procedures were explained by our team, physicians declined to participate because many of their clients did not speak or read English or French well (n = 26), they were too busy (n = 12), or had other reasons (n = 26), such as being away or on maternity leave during the time data were to be collected.

There was also some attrition that affected the cooperation rate (n = 254) for similar reasons. Nineteen (7%) physicians did not meet our eligibility requirements or our team was unable to contact the physician (46%). Other common reasons for attrition in the cooperation rate was that some physicians (21%) reported that administering the survey was not feasible because appropriate approval in working with First Nation patients had not been sought by the practice, or stated that the surveys would take too long to complete.

Half the 792 family physicians were female and just under half (45.3%) were 65 years or older (Table 3). The majority (71.6%) were born in Canada with an overwhelming percentage (90.3%) indicating they were self-employed and working in group practices. Providers worked on average 41 hours per week, though there was wide variability in their reported work hours. Participating practices were from a variety of geographic locations, with just under one-third reporting they delivered services in an inner city. Just under 40% of physicians perceived that the number of elderly patients from their panel was above national average. Over half reported being open after 6 pm with fewer practices having connections to either lab (29.8%) or x-ray (19.3%) facilities.

**Table 3:** **Respondent characteristics**

**Provider’s characteristics**	**n = 792**
Sex, Female: n (%)	393 (49.6)
Age: n (%)	
Under 44	19 (2.4)
45-64	408 (52.2)
65+	354 (45.3)
Born in Canada: n (%)	
Yes	563 (71.6)
Self-employed or salaried: n (%)	
Self-employed	708* (90.3)
Average hours worked per week: Mean (SD)	40.7 (12.7)
**Practice characteristics**	
Solo or group practice: n (%)	
Group practice	708 (90.3)
Geographic profile: n (%)	
Inner city	236 (30.1)
Suburbs	136 (17.3)
Small town	153 (19.5)
Mixed urban–rural	107 (13.6)
Rural	152 (19.4)
Patients above national average: n (%)	
Elderly	305 (38.6)
Disadvantaged	226 (28.6)
Ethnic minority	157 (19.9)
Extended hours: n (%)	
Open after 6 pm (at least once a week)	432 (54.9)
Open weekends (at least once a month)	303 (38.9)
Access within practice/centre to: n (%)	
Lab facilities	236 (29.8)
X-ray facilities	152 (19.3)
**Patient characteristics**	8,332 (7,172 patient experiences + 1,160 patient values)
Sex, Female: n (%)	N = 5,447 (66.7)
Age: n (%)	
18-30	957 (12.1)
31-44	1,678 (21.2)
45-64	3,320 (41.9)
65+	1,971 (24.9)
Education: n (%)	
No qualifications, pre-primary, primary, or lower secondary	761 (9.4)
Upper secondary education (grades 10–12)	2,587 (31.9)
Post-secondary education (includes college, undergraduate)	4,765 (58.7)
Presence of chronic condition(s): n (%)	
Yes	4,547 (55.5)
General health status: n (%)	
Very good	1,859 (22.6)
Good	4,249 (51.6)
Fair	1,797 (21.8)
Poor	332 (4.0)

Note. All characteristics are self-reported. All categorical data do not add up to 100% due to missing data. *14 of these 708 respondents indicated they are both salaried and self-employed.

The majority of patient respondents were female (66.4%). Many patients were between the ages of 45–64 years (41.9%). Four out of every 10 patient respondents reported not having a post-secondary education. The majority of patients reported their health as very good or good (73.7%) and had a chronic condition (55.6%).

## Discussion

Initial response rates in our study varied from province to province but were generally low across Canada. This response rate for the QUALICO-PC arm in Canada was similar to rates found in other countries such as Australia [37] (ranged from below 1% to 14.5%) and New Zealand (12.2%). Indeed, the difficulties obtaining acceptable response rates from family physicians for survey participation is a universal challenge. However, our results suggest that once family physicians agree to participate in a survey, their participation and cooperation rates are high.

There are some lessons learned from in the recruitment of family physicians for multijurisdictional studies. In Canada, where funding was obtained from multiple sources and varied by province, the low payment to participating physicians made it possible for all 10 provinces to participate. Yet, the low response rate is reflective of the minimal amount of resources available for recruitment in this pan-Canadian study. This amount may seem like a high amount for participation in a study; ethics boards would suggest even higher amounts may unnecessarily influence potential participants to ‘voluntarily enter the study’ [38]. From a small business viewpoint, this amount of money could be seen as less than a token of appreciation since it would not cover even half the costs of disruption to their businesses. Offering a larger payment in appreciation of the disruption to their practices might have improved the initial response rate but likely not enough to meet the targeted sample size at the provincial level. Past work has shown that a monetary incentive significantly improves physicians’ response rates [39] yet, little remains known about how much monetary incentive is needed to achieve adequate response rates (e.g., ≥50%) [40] amidst the known trend of decreasing response rates in this population [41,42]. There are likely other factors that also influence family physician participation in studies such as the lack of time in building relationships with the front office staff. A fine balance between resources available and payment for participation needs to be carefully assessed.

A low response rate from family physicians is, in part, due to structural challenges. Without a central source for such data, researchers and others (including provinces) must individually seek out family physicians and their patients. Other sectors of the health care system are required to regularly report on specific information to a provincial and often a pan-Canadian repository that is made available to them for the purpose of quality improvement and others for the purpose of planning health services and research. Examples of these data include the Discharge Abstract Database from acute care and the Resident Assessment Instrument from long term and home care. Not only is does each organization required to report data have specific resources to obtain the necessary data but at the pan-Canadian level, the Canadian Institute for Health Information [43] manages the quality of the data. Currently primary care has no such required reporting requirements or the infrastructure to produce any information using such data.

Another structural challenge in collecting data from primary care is that no province keeps an up-to-date list of practicing family physicians. This is somewhat surprising given the amount of public money spent on physician services by government. What we did find is that the degree to which provinces have accurate family physician lists varies. Some of the smaller provinces update their lists every few years whereas in the larger provinces, accuracy of the lists exists at a health authority or other organizational (e.g. division of family practice in British Columbia) level.

As with any study, this work has limitations. Our recruitment strategy was to contact individual physicians to participate in our study because no list exists to recruit at a practice level. Using the lists of family physicians we could obtain, it is likely that our denominator used for calculating the response rate is larger than who was actually eligible to participate (only one family physician per practice who did not have a specialized practice). Although we had limited resources to carry out recruitment, more highly resourced and similar surveys (e.g. Commonwealth Fund International Health Policy Survey [13], Commonwealth Fund International Health Policy Survey of Sicker Adults [44]) also have poor response rates (albeit higher than ours). Cross country surveys aimed at family physicians may be able to increase its response rates by putting more effort (and resources) into creating situations which could increase the face validity of the study [45]. Finally, given the low response rate within provinces, it is not possible to compare primary care practices at a provincial level. However, useful analyses looking for associations between the quality of primary care and practice and physician characteristics can still be undertaken, similar to what has been done by the Commonwealth fund [13,44,46].

We also note that the recruitment methods varied across provinces. Regardless of trying to recruit using a randomized sample or a census approach, the response rate remains low across all provinces. It is possible that a more involved and longer recruitment approach such as what was used in Québec could increase participation in future practice-based primary care studies. In order to implement this across Canada, more resources such as an up-to-date physician list and full time staff to conduct the study would be needed.

Despite these limitations and overall low response rate, the data collected through the Canadian arm of QUALICO-PC represents the largest dataset on the quality and organization of primary care data in Canada. Through our collective recruitment efforts we have the largest number of cooperating practices, physicians, and patients compared to any other country participating in QUALICO-PC. These data can tell us about important patient experiences and values among those who have access to primary care and how practice and provider characteristics might impact patient outcomes such as activation, the degree to which patients become engaged to manage their own care [47].

## Conclusion

Generally there is a need to have better information in the area of primary care. Challenges that need to be addressed in how to obtain better information include increasing response rates from primary care providers and using an appropriate sampling strategy that acknowledges the shift to team-based care and increasingly different models of care (e.g. nurse practitioner practices). Moreover, a more coordinated effort to gather this information using short, valid surveys is needed in Canada but also internationally. Even though most family physicians operate as small businesses, they could be supported to routinely submit data through a collective effort and provincial mandate. The groundwork in setting up pan-Canadian collaboration in primary care has been established through this study. Future work to attain external validity is now needed. Establishing an information structure that routinely collects primary care data will be key to developing evidence-informed policy and delivery of health services.

## Abbreviations

QUALICO-PC: Quality and Costs of Primary Care
PHC: Primary Health Care
OECD: Organization for Economic Co-operation and Development
EU: European Union
PRA: Practice Survey
FPS: Family Physician Survey
PES: Patient Experiences Survey
PVS: Patient Values Survey
PHN: Personal Health Number
